# Shaken Adult Syndrome: Defining a New Traumatic Entity with an Evidence-Based Approach

**DOI:** 10.3390/diagnostics16020319

**Published:** 2026-01-19

**Authors:** Fabio Del Duca, Gianpietro Volonnino, Biancamaria Treves, Alessandra De Matteis, Nicola Di Fazio, Raffaele La Russa, Paola Frati, Aniello Maiese

**Affiliations:** 1 Department of Biomedicine and Prevention, University of Rome “Tor Vergata”, Via Montpellier 1, 00133 Rome, Italy; 2 Department of Medicine, UniCamillus-Saint Camillus International University of Health Sciences, 00131 Rome, Italy; 3 Department of Anatomical, Histological, Forensic and Orthopedic Sciences, Sapienza University of Rome, Viale Regina Elena 336, 00161 Rome, Italy; 4 Department of Life Sciences, Health and Health Professions, Link Campus University, 00165 Rome, Italy; 5 Department of Clinical Medicine, Public Health, Life Sciences, Environmental Sciences, University of L’Aquila, 67100 L’Aquila, Italy

**Keywords:** shaken adult syndrome, domestic violence, abusive head trauma, head trauma, subdural hemorrhage, retinal hemorrhage, diffuse axonal injury

## Abstract

Major traumas result from the application of multiple force components that, in adulthood, can lead to high mortality and morbidity. In forensic practice, pathological consequences arising from the rapid flexion–extension of an adult victim’s soma are observed, with typical intracranial and ophthalmological findings. The totality of these findings allows for a contribution to the definition of the Shaken Adult Syndrome (SAS). A comprehensive review, employing the PRISMA methodology, was conducted on international works pertaining to SAS. This resulted in the identification of six scientific papers, which were analyzed separately. It emerged that, for the diagnosis of SAS, the same diagnostic triad as Shaken Baby Syndrome is valid, comprising subdural hemorrhages, retinal hemorrhages, and encephalopathy. This syndrome appears to encompass a broad spectrum of pathological conditions, ranging from whiplash to diffuse axonal injury (DAI). At the conclusion of this work, we proposed a diagnostic flowchart that allows for suspected predictive diagnosis of SAS, both in live patients presenting to emergency medical services and in post-mortem cadavers. For this purpose, the collection of anamnesis and circumstantial data, the detection of external injuries, and the execution of cranial CT scans will be essential. Ultimately, microscopic examinations of the brain with specific immunomarkers and of ocular structures will enable the identification of pathognomonic findings for SAS.

## 1. Introduction

Shaken Adult Syndrome (SAS), known as inflicted head injury by shaken trauma (IHI-ST), represents a severe form of non-accidental traumatic brain injury, resulting from the transmission of mechanical forces, frequently external, to the body of an adult victim. This condition typically arises when an individual is subjected to violent shaking, producing a constellation of neurological injuries reminiscent of those observed in infants, such as subdural hematomas and retinal hemorrhages [1].

The underlying biomechanical mechanisms involve rapid acceleration–deceleration forces, capable of inflicting significant cerebral trauma. Despite anatomical and physiological differences between adults and infants, the hypothesis is that adults remain susceptible to serious injury through similar violent mechanisms [2,3].

Evidence from clinical literature indicates that the presentation of shaking-related injuries in adults may parallel that seen in pediatric cases [4,5,6,7]. Neurological signs such as confusion, hemiplegia, and diplopia may be present, though they may be misattributed to other etiologies. As a result, SAS in adults might be underdiagnosed, leading to an underappreciation of the broader implications of interpersonal and domestic violence.

In geriatric populations, particularly among frail individuals with multiple comorbidities, intracranial hemorrhages and parenchymal injuries due to shaken forces may occur in the absence of overt external trauma [8,9,10,11], complicating forensic recognition and clinical assessment [12].

Beyond the immediate physical injuries, long-term pathological consequences may include cognitive decline, psychological trauma, and persistent neurological deficits [13,14,15,16,17,18]. Research underscores that, similar to the pediatric population, the structural and functional vulnerabilities of the adult brain can result in profound internal damage when exposed to high-velocity shaking [19,20,21,22,23,24].

Although episodes of adult shaking may occur in a variety of settings, the social and preventive implications mirror those associated with Shaken Baby Syndrome [25,26,27,28,29,30,31,32,33,34,35,36,37,38,39,40]. Educational initiatives aimed at increasing public and professional awareness of the dangers of violent shaking and at promoting non-violent conflict resolution have been advocated as essential measures to reduce the incidence of such trauma. This includes informing both potential victims and perpetrators about the risks and long-term consequences of violent behavior [41]. Despite the clinical symptoms being clearly depicted in the current literature, autopsy findings are less well-known by the scientific community. In fact, the literature on Shaken Adult Syndrome is almost exclusively composed of case series. There is a need for a work to collect and synthesize the evidence from the individual contributions in the literature.

To date, the pathophysiological mechanism of shaking in infants, which results in a syndrome known as shaken baby syndrome, is well established. This condition is often associated with episodes of child abuse; for this reason, shaken baby syndrome has been the subject of numerous studies in the literature, and diagnostic pathways have been developed to enable its early identification. Similar shaking of the head can produce, through the same pathogenic mechanism, a comparable clinical picture in adults; however, this entity has not yet been investigated to the same extent in the literature, despite its relevance.

In conclusion, while Shaken Adult Syndrome shares key pathological features with its pediatric counterpart, awareness and recognition remain critically insufficient. Greater clinical vigilance, improved identification protocols, and a societal shift toward prevention through education and support are essential to addressing this under-recognized form of domestic violence and safeguarding vulnerable populations. The aim of the study is to provide a comprehensive overview of the forensic diagnostic challenges in shaken-related trauma, focusing on the key clinical, radiological, and pathological findings, the main differential diagnoses, and the medico-legal implications for accurate case assessment.

## 2. Materials and Methods

A comprehensive review of the updated literature was carried out according to the Preferred Reporting Items for comprehensive Review (PRISMA) standards [42], as previously reported in previous studies [43,44,45,46,47]. The review protocol was developed a priori to ensure transparency and reproducibility, and all methodological steps were performed in compliance with PRISMA standards.

Although the protocol was not registered in PROSPERO, the methodological framework strictly followed the PRISMA 2020 checklist and flow diagram, ensuring standardized reporting of identification, screening, eligibility, and inclusion stages.

Eligible studies were selected according to the following inclusion criteria:

- Articles published in peer-reviewed journals between January 1990 and September 2025;
- Studies written in English;
- Studies involving adult human subjects (≥18 years) diagnosed with Shaken Adult Syndrome or adult shaken head trauma;
- Articles presenting original data (case reports, case series, or observational studies).

Exclusion criteria included:

- Non-human studies, pediatric populations, reviews, conference abstracts, editorials, and non-English papers;
- Studies lacking diagnostic, histopathological, or imaging evidence consistent with shaking mechanisms

Three electronic databases were systematically searched: PubMed, Scopus (ScienceDirect), and Google Scholar. The last search was conducted in September 2025. Reference lists of included articles were also manually screened to identify additional relevant studies. The following Boolean search string was used across all databases: (“Shaken Adult Syndrome” OR “Adult Shaken Head Trauma” OR “Shaking Syndrome” OR “Whiplash”) AND (“adult” OR “elder”) AND (“autopsy” OR “post-mortem” OR “postmortem”).

Searches were performed in all fields (title, abstract, and keywords). The results were exported in nbib format and managed using Zotero 6.0.30 for reference organization and deduplication.

**Figure 1:**
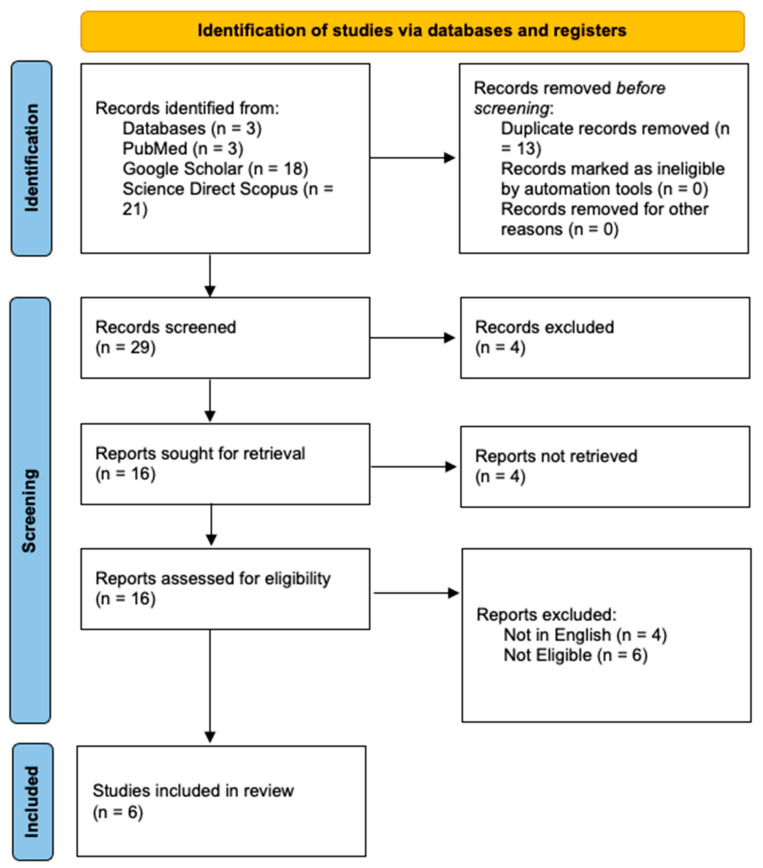
PRISMA Standard has been applied to database research.

Two independent reviewers (F.D.D. and N.D.F.) screened titles and abstracts for eligibility. Full texts of potentially relevant articles were then retrieved and assessed according to the inclusion and exclusion criteria. Disagreements were resolved by consensus or by a third reviewer (G.V.). No article from backward snowballing was retrieved. The selection process is detailed in the PRISMA 2020 flow diagram (Figure 1).

Data were extracted from each included study using a standardized data collection form. Extracted variables included: year and country of publication, study design, patient demographics (age, sex), clinical history, cause and mechanism of trauma, neuroimaging findings, autopsy results, histological and immunohistochemical findings (particularly β-amyloid precursor protein expression), and ocular findings.

Given the qualitative nature of the included studies (mainly case reports and case series), a formal risk-of-bias assessment was not applicable. Nevertheless, methodological quality and internal validity were appraised through careful evaluation of diagnostic consistency, data completeness, and concordance with established neuropathological criteria for traumatic brain injury.

The extracted data were synthesized descriptively. The results were summarized in tables highlighting demographic characteristics, clinical presentation, imaging features, and histopathological findings. A narrative synthesis was then performed to integrate findings and identify recurring diagnostic patterns across studies.

### Risk of Bias

This systematic review targets articles published within the last thirty five years, employing specific research methods utilizing a select few keywords.

Given the qualitative nature of the included studies, which consist primarily of case reports and case series, a formal risk-of-bias assessment using traditional tools designed for randomized trials (e.g., RoB 2) was not applicable. Key study limitations include the absence of prospective protocol registration (e.g., in PROSPERO), which should not be applied because this paper is not a systematic review. Additionally, while the review timeframe was consistently defined as January 1990 to September 2025 to encompass foundational and recent literature, this broad span may introduce heterogeneity in diagnostic practices over time. These factors collectively rate the overall certainty of evidence, highlighting the need for higher-quality observational studies to validate SAS diagnostic criteria

## 3. Results

After the complete database screening, a total of seven cases were retrieved from case reports and case series. All of them regards adult, and only in one case was an old man involved (>65 years old). Mean age was 53.2 years old ± SD 18.89 (s^2^ = 357.2, IQR = 34, IQ_1–3_ = 37–71), which shows no age prevalence (Table 1).

Table 2 shows the different clinical presentations of shaken syndrome in adults, all caused by a single underlying mechanism. In every case, the head was subjected to rapid acceleration–deceleration forces, either indirectly by shaking the shoulders or body (indirect shaking syndrome), or directly by shaking the head itself. Only one individual survived the assault, while six out of seven cases resulted in rapid death (See Table 3).

### Descriptive Review of the Study’s Methodology

In the study by Carrigan et al. [1], a case is described involving a 34-year-old woman who presented to the emergency department claiming she had fallen down the stairs. Upon arrival, she exhibited the triad of subdural hematoma, retinal hemorrhages, and fingertip-shaped bruising. The woman had also ingested alcohol. Only after 48 h and following repeated questioning did she admit that she had, in fact, been the victim of domestic violence rather than a simple fall. At admission, her Glasgow Coma Scale score was 13/15 due to a confused mental state, but it rose to 15/15 within the following 24 h. She experienced post-concussion symptoms for around two weeks, reported episodes of vomiting, and complained of persistent blurred vision. Her visual acuity was reduced to hand movements in the right eye and finger counting in the left. Clinical examination revealed fingertip bruising over her back, buttocks, and arms, as well as scalp hematoma, a frontal abrasion, periorbital bruising, and epistaxis. Although radiographic skull and facial bone examinations were unremarkable, CT imaging revealed a small left temporal subdural hemorrhage with associated edema. Ophthalmological evaluation demonstrated bilateral retinal and preretinal hemorrhages along with macular bleeding.

The case reported by Jennian F. Geddes [48] involves a Palestinian man who died after three days of interrogation and assault by Shin Bet officers. External examination revealed bruising over the anterior thorax bilaterally, while the face, neck, and scalp showed no external trauma. Upon craniotomy, cerebral edema and subdural hemorrhage were identified. Microscopic brain analysis showed edema, subdural bleeding, and diffuse axonal injury (DAI), confirmed via amyloid precursor protein (APP) immunohistochemistry. Histological slides described “shrinkage beads of nerve fibers”, particularly within the corpus callosum. Microscopic eye examination revealed retinal hemorrhage.

Amir A. Azari [49] presented two reports. The first was of a man in his fifties found dead after an assault in which, according to an eyewitness, he was grabbed from behind and shaken back and forth three to four times. External examination revealed no head trauma. Craniotomy revealed a unilateral right subdural hemorrhage and cerebral edema without signs of herniation. Blood toxicology showed alcohol. Standard histology confirmed subdural bleeding. Immunohistochemical staining for APP showed bilateral positivity within the centrum semiovale and dorsal midbrain white matter. Ocular microscopy demonstrated retinal hemorrhages, vitreous hemorrhage, macular folds, extraocular muscle hemorrhages, and pathological alterations of the optic nerve, including edema and subdural/subarachnoid hemorrhage of the nerve sheath.

The second case described a man in his sixties found unconscious at home; two friends attempted resuscitation by repeatedly shaking him. External examination showed various bruises on the chest and lower limbs, likely self-inflicted, with no cranial injuries or fractures. Craniotomy revealed bilateral subdural hemorrhage, cerebral edema, Duret midbrain hemorrhages, and transtentorial herniation. Microscopy confirmed subdural and focal subarachnoid hemorrhages with areas of tissue necrosis. APP staining showed nonspecific positivity. Toxicology showed alcohol. Ocular microscopy showed retinal hemorrhages, vitreous hemorrhage, macular folds, and hemorrhages of the extraocular muscles and optic nerve sheath.

Kenji Ninomiya [51] reported an apparent drowning case involving a ~40-year-old man swept away while fishing on a rocky shore. His body was recovered adrift hours later. Postmortem CT showed subarachnoid hemorrhage (SAH). External examination revealed no head injuries. Lungs were overinflated, weighing 820 g and 1040 g, with massive frothing in airways and pleural effusion of 60 mL left and 100 mL right. Craniotomy showed brain edema and SAH within the interhemispheric fissure and parieto-occipital convexity, as well as small caudate nucleus hemorrhages. Conventional microscopy showed multiple mesencephalic foci; APP staining was negative. Toxicology was negative. The traumatic midline SAH and DAI indicated rotational shearing forces from the whirlpools rather than direct impact with rocks.

**Figure 2:**
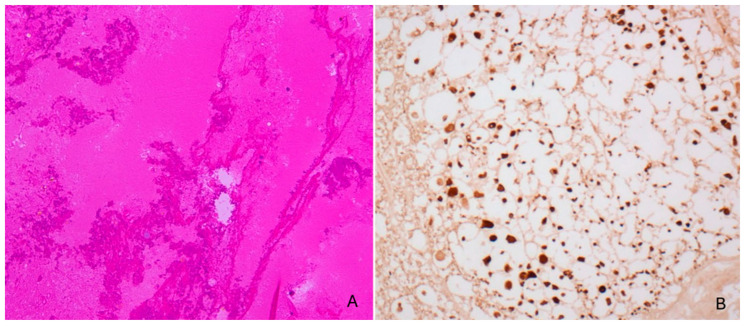
From Zhendong Li et al. [50] (**A**) Cerebral hemorrhage visible, hematossilin and eosin staining (**B**) immunohistochemistry (anti APP) shows staining positivity of β-amyloid precursor protein. (magnification 20×) into cerebral cells.

Zhengdong Li et al. [50] described a detainee shaken over 20 times within 12 h by fellow prisoners, captured on CCTV (Figure 2). He developed gait instability and entered a coma before CT revealed cerebral edema, midline shift, and left temporal subdural hemorrhage. Decompressive craniectomy and hematoma evacuation were performed, but he died ten days later. Autopsy confirmed cerebral edema, subdural bleeding, and DAI via APP immunostaining.

Bugelli et al. [12] reported an 82-year-old woman found unresponsive at home with a GCS of 3. CT revealed right parieto-temporal and left parieto-occipital subdural hemorrhages with midline shift, superimposed on chronic ischemic encephalopathy. No thoracic or abdominal injuries were present. She died after four days. External examination showed multiple bruises on upper and lower limbs compatible with forceful grabbing or repeated blows, as well as bruises on the chest, face, head, and neck, with minimal or absent cranial contusions. Craniotomy showed bilateral subdural hemorrhages. Microscopy dated these to 5–7 days due to granulation tissue, erythrocyte breakdown, siderophages, fibroblast infiltration, and capillary neovascularization. Severe edema with large pericellular and perivascular spaces was also observed. Toxicology was negative for alcohol or drugs.

## 4. Discussion

Based on the available literature, Shaken Adult Syndrome can be identified in both living patients and deceased individuals. It is necessary to define the diagnostic steps required to reach an accurate diagnosis of this syndrome while excluding alternative forensic diagnoses.

In all case studies, where a patient or deceased individual presents with suspicious injuries, it is imperative to conduct a thorough investigation of the medical history and/or circumstantial data to exclude an episode of violence [1,52].

Although during the initial assessment the patient may deny experiencing violence, it is essential to re-evaluate the patient’s history in the subsequent days, as they may later disclose the violence suffered. This aspect is crucial, as it may lead the patient to file a complaint or seek legal or social assistance [53].

Furthermore, in many countries, including several US states and Italy, physicians are legally required to report injuries resulting from assault in any patient presenting to the emergency department. This creates an ethical and legal dilemma for emergency physicians, who are obliged to report suspected domestic violence even in the absence of explicit patient disclosure, which may only occur after several days. Unfortunately, victims of domestic violence are often discharged the same day, resulting in the loss of an important diagnostic window for Shaken Adult Syndrome [54].

Physical, cerebral, and ocular injuries can worsen over time and may be fatal; therefore, a structured emergency-department follow-up can support a more accurate investigation and help uncover violence, preventing further episodes as in pediatric protocols. In deceased cases without a history, scene investigation, circumstantial review, and witness interviews are essential. Typical findings include bruises (sometimes with fingertip marks) and abrasions, and subdural hematomas without external head contusions, suggesting shaking-related acceleration–deceleration and rotational forces rather than direct impact [55].

Neuroimaging, specifically brain CT scans, whether conducted ante-mortem or post-mortem, typically reveals unilateral or bilateral subarachnoid hemorrhages, predominantly located in the temporo-parietal regions. These are often associated with a midline shift secondary to increased intracranial pressure [51].

All autopsy examinations generally revealed characteristic findings, particularly at the cerebral level, such as unilateral or bilateral subdural hemorrhages, often associated with cerebral edema. Additional findings may include small subarachnoid hemorrhages, focal tissue necrosis, and signs of brain herniation.

However, in certain cases, pathological alterations may also involve other organs. For example, in the case report by Kenji Ninomiya [51], the decedent suffered injuries not only from head shaking but also from an asphyxial mechanism due to drowning. The lungs were markedly overinflated, weighing 820 g and 1040 g, respectively, with massive froth present in the upper and lower airways. Pleural effusions of 60 mL (left) and 100 mL (right) were also observed. In such instances, it is crucial to establish the predominant mechanism responsible for death.

The next diagnostic step involves microscopic examination of the brain and ocular structures. Brain histology confirms macroscopic findings of subdural hemorrhage and edema, and enables assessment of diffuse axonal injury (DAI) through sampling of multiple brain regions. Conventional staining techniques reveal “shrinkage beads” of nerve fibers within the white matter. Immunohistochemistry for amyloid precursor protein (APP) is typically positive, confirming axonal damage [56,57].

Microscopic examination of ocular tissues demonstrates retinal hemorrhages and macular folds. Retinal hemorrhages [1] are generally bilateral, symmetrical, and may be intraretinal, preretinal, or subretinal. The most affected regions are those with maximum vitreoretinal adhesion, including the ora serrata, posterior pole, and perivascular areas.

Additionally, the optic nerve is frequently involved, with findings such as papilledema and subdural or subarachnoid hemorrhages. The extraocular muscles are often affected as well, frequently presenting with hemorrhages [49].

A crucial differential diagnosis is Terson Syndrome [58], which is defined as a vitreous hemorrhage associated with subarachnoid hemorrhage. However, its current clinical application encompasses any coexistence of intraocular and intracranial hemorrhage. In contrast, Shaken Adult Syndrome is characterized by the additional presence of hemorrhages within the extraocular muscles, sclera/episclera, and retina at the ora serrata, as well as macular folds and retinal hemorrhages—findings absent in Terson Syndrome.

Reported cases are heterogeneous but share a common acceleration–deceleration mechanism, namely shaking. This mechanism is responsible for the development of cerebral edema and subdural hemorrhage, leading to increased intracranial pressure and subsequent brain herniation.

A critical aspect of this pathophysiology is the frequent absence of direct cranial trauma. Shaking does not depend on impact with an external object. Indeed, the main hypothesis is that it alone generates the forces necessary to cause subdural hemorrhage. The shaking may be performed by gripping either the shoulders or the head directly.

**Figure 3:**
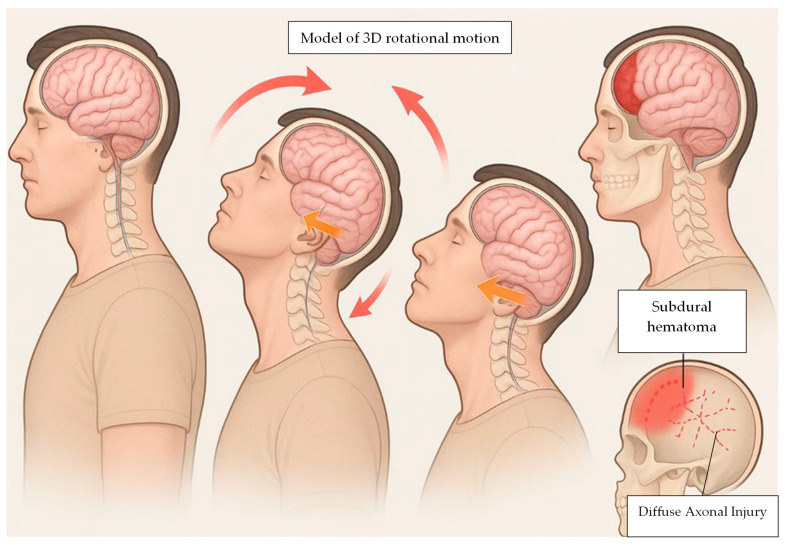
A rapid 3D-rotational motion induces concussion and internal cerebral damage.

When describing the pathogenetic mechanism, the predominant feature is rapid flexion–extension and rotational movement of the head, while the torso remains relatively stationary (Figure 3). In some cases, the torso acts as a pivot, whereas in others the head is shaken directly [59].

This mechanism can result from another individual’s mechanical action or from natural forces, such as wave motion, as described in the aforementioned case report. Identified risk factors include alcohol ingestion and significant physical differences between the victim and aggressor in terms of height, age, or muscular strength.

These forces produce diffuse axonal injury in the white matter and hemorrhages within midline brain structures.

It is important to emphasize that all the literature sources considered in this article showed overall agreement both on the macroscopic and microscopic autopsy findings and on the shaking mechanism that produces such injuries, which appears to be pathognomonic of Shaken Adult Syndrome.

In conclusion, a diagnostic proposal is needed to facilitate the early and accurate diagnosis of Shaken Adult Syndrome in both living patients and post-mortem examinations.

## 5. Conclusions

**Figure 4:**
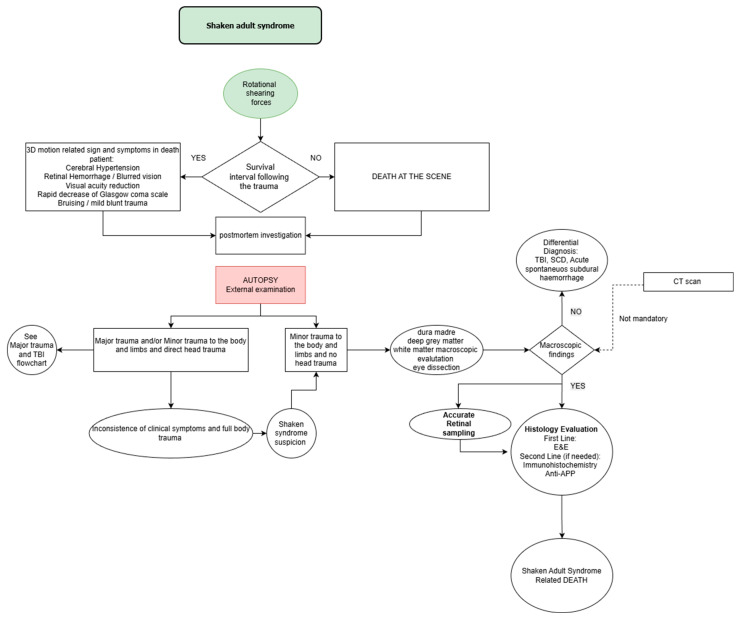
Shaken Adult Syndrome diagnostic flowchart.

The diagnosis of Shaken Adult Syndrome (SAS) represents a still little-known yet distinct and clinically significant condition, characterized by the triad of subdural hemorrhage, retinal hemorrhages, and optic nerve sheath hemorrhages. Those findings are often associated with diffuse axonal injury (DAI). The triad described above is, however, too often underestimated. In adult individuals, this type of lesion may occur in various contexts—such as domestic violence, physical assault, or sudden accidental movements—and can develop even in the absence of any clear cranial trauma. In this study, we propose a diagnostic flowchart aimed at guiding both clinicians and forensic pathologists towards the recognition of a syndrome that is at once subtle and severe. Starting with the assessment of an individual exposed to rotational forces to the head, it is necessary to investigate possible pre-mortem symptoms. Regardless of whether the person survives, a proper external examination must be carried out to exclude major external trauma. Subsequently, an appropriate autopsy examination (see Figure 4) and histological confirmation are necessary to arrive at a diagnosis of Shaken Adult Syndrome.

A multidisciplinary clinical and medico-legal approach will therefore be necessary. To this end, a thorough examination of the brain is essential, involving a cranial CT scan in the living patient to detect subdural hemorrhages or signs of cerebral edema, followed by macroscopic and microscopic examination with immunohistochemical analysis in the deceased, in order to highlight diffuse axonal injuries and vascular damage consistent with shaking mechanisms. At the same time, examination of the retinal structures is mandatory. This can be performed through a fundus oculi examination in living patients or by microscopic evaluation of the retina, optic nerve, and extraocular muscles in the cadaver. Retinal and optic nerve sheath hemorrhages, particularly when bilateral and symmetrical, represent highly suggestive findings of SAS and may serve as discriminating elements in differential diagnosis, especially in distinguishing SAS from Terson’s syndrome. For these reasons, and with a view to future developments, it would be necessary to implement ocular diagnostic tools comparable in diagnostic value to immunohistochemical staining techniques used for cerebral analysis. The recognition of SAS has significant clinical, forensic, and legal implications. Identifying the underlying traumatic mechanism through a rigorous diagnostic process carries considerable social importance, as it enables the protection of vulnerable individuals who are victims of violence. For this reason, future research should promote interdisciplinary collaboration among clinicians, neurologists, ophthalmologists, radiologists, forensic pathologists, and biomechanical engineers. Specifically, in the future, it would be advisable to collect additional data and to disseminate a diagnostic algorithm that enables proper diagnostic and therapeutic management of the syndrome. The consolidation of Shaken Adult Syndrome within the pathophysiological framework of traumatic brain injuries will contribute to preventing further episodes of abuse and to strengthening both diagnostic awareness and medico-legal protection [18,19].

## Figures and Tables

**Table 1:** Demographic analysis of the selected cohort.

Reference	Year	Country	Type of Paper	Sex	Age
Jennias F. Geddes [48]	1995	Israel	Case Report	M	-
Carrigan T.D. et al. [1]	1999	UK	Case report	F	34
Amir A. Azari et al. [49]	2013	N/A	Case Series	M	50
				M	60
Zhengdong Li [50]	2021	China	Case report	M	-
Kenji Ninomiya [51]	2023	Japan	Case report	M	40
Bugelli et al. [12]	2023	Italy	Case report	F	82

**Table 2:** Clinical presentations of shaken syndrome in adults.

Reference	Age	Clinical History	Outcome	Type
Jennias F. Geddes [48]	-	-	alive	Died 3 days after interrogation and assault
Carrigan T.D. et al. [1]	34	-	death	Domestic violence (24 h delay for ED admission)—Initially referred fallen down stairs after Alcohol ingestion
Amir A. Azari et al. [49]	50	Arterial hypertension	death	Assault
	60	Alcohol abuse	death	Attempted to resuscitate through “*vigorous shaking*” by the shoulders. Shaken by two adults while unconscious
Zhengdong Li [50]	-	-	death	Drowning—was washed away by a wave during recreational fishing
Kenji Ninomiya [51]	40	-	death	Prison inmate. Shaken repeatedly (more than 20 times) while in a sitting posture within 12 h by other prisoners. State of coma after a few hours. A decompressive craniectomy and intracerebral hematoma evacuation were performed, but he died after 10 days The perpetrator directly held the victim’s head in his hands and shook it violently.
Bugelli et al. [12]	82	Widespread cortical atrophy, progressive cognitive impairment, depression, hypertension, hypercholesterolemia, osteoporosis, megaloblastic anemia and mild renal insufficiency	Death 4 days after hospitalization	Domestic violence

**Table 3:** The macroscopic and microscopic findings are homogeneous across all cases. Here, the findings of the individual cases are described, and the causes of death are compared.

Reference	Imaging	Timing from Assault	Autoptic Macroscopis Finding	Histological Cerebral Findings	Histological Eye Findings	Cause of Death
Jennias F. Geddes [48]		Died 3 days after interrogation and assault	Extensive bruising was present on both sides of the anterior chest, with no evidence of injury to the neck, face, or scalp. There was cerebral edema and a unilateral subdural hematoma causing distortion of the underlying brain.	Diffuse axonal injury (DAI). Diffuse pattern in the white matter, especially in the corpus callosum, where there are ‘shrinkage beads’ of nerve fibers.	retinal hemorrhages	Intracranial hypertension due to cerebral hemorrhage
Carrigan T.D. et al. [1]	CT brain—small left temporal subdural hemorrhage with near edema	3 days	/	/	/	N/A
Amir A. Azari et al. [49]	/	/	No external physical findings were present on the face, head, or inner aspect of the scalp. Unilateral subdural hemorrhage was present over the right dorsal cerebrum. Minor features of gross brain swelling without herniation were evident.	Histological analysis of brain tissue showed minor foci of acute subarachnoid hemorrhage in association with the gross subdural bleeding. Amyloid precursor protein immunohistochemistry results were positive in a bilateral distribution in the white matter of the centrum semi ovale and dorsal midbrain.	Histological studies on both eyes revealed extensive subdural and subarachnoid hemorrhages within the optic nerve sheath. Blood was also present within the vitreous, subretinal space, inner nuclear layer, outer plexiform layer, and outer nuclear layer, with involvement of the ora serrata in both eyes. Additional bilateral findings included swelling of the optic nerve heads, macular folds, and extraocular muscle hemorrhage.	subdural/subarachnoid hemorrhage and Diffuse axonal injury (DAI)
			2.0 cm area of erythema over the superior margin of the right temporalis muscle, bilateral, subdural hemorrhage. Cerebral edema with right transtentorial herniation and secondary Duret midbrain hemorrhage were present.	Tissue necrosis and focal subarachnoid hemorrhage in association with the gross subdural hemorrhage. Immunohistochemical staining for amyloid precursor protein showed nonspecific positivity.	Ophthalmic examination revealed bilateral subdural and subarachnoid hemorrhages within the optic nerve sheaths. Blood was also identified in the vitreous, as well as in the inner and outer retinal layers and the subretinal space of both eyes. Retinal hemorrhages extended to the ora serrata bilaterally. Prominent macular folds were observed in both eyes, along with hemorrhage involving the extraocular muscles and episclera.	Transtentorial herniation
Zhengdong Li [50]	Left temporal occipital lobe intra-cerebral hemorrhage, hemoventricle, and brain herniation. Optical nerve bilateral distortion.		left temporal occipital lobe hemorrhage, hemoventricle, left temporal subdural hemorrhage, cerebral edema, and formation of cerebral softening foci.	Broad spectrum of pathological changes in deep white matter, intraparenchymal hemorrhage.		subdural bleeding and DAI
Kenji Ninomiya [51]	SAH on the anterior side of the longitudinal fissure and the surrounding cerebral sulcus.	/	No evidence of external examination of mechanical trauma was found on the surface of the body. Lungs were overinflated [left 820 g; 1040 g]. Marked frothing was present throughout the upper and lower airways. Pleural effusions measuring 60 mL on the left and 100 mL on the right were noted. The brain [1520 g] exhibited swelling and congestion. Subarachnoid hemorrhage was present within the interhemispheric fissure and over the convexity of the parietal-occipital lobe. Small hemorrhages were observed in the left caudate nucleus, putamen, external globus pallidus, hypothalamus, septum pellucidum, midbrain, pons, and dentate nucleus.	Macroscopic hemorrhages were present in hematoxylin and eosin (HE)-stained sections of the brain. Many microscopic hemorrhagic spots were also found in the midbrain, pons, and other white matter of the whole brain. Immunohistochemical staining for the β-amyloid precursor protein (APP) was negative.	/	midline SAH and DAI
Bugelli et al. [12]	Right parieto-temporal subdural hematoma, 21 mm thick, compressing the right lateral ventricle and third ventricle, causing a midline shift of about 9 mm. Additional findings were represented by a left temporo-occipital SDH, 8 mm thick, right temporo-basal intraparenchimal hemorrhages, and blood in the fourth ventricle. No fractures or other traumatic injuries of the internal organs were found in the thorax or abdomen.		Multiple bruises were found on the head and face, neck, thorax, and upper and lower extremities.	A frontoparietal hematoma, with a diameter of 3.5 cm, associated with a bilateral subacute SDH, approximately 2 cm thick, with fresh coagulated blood between the arachnoid and dura mater.	The subdural hematomas (SDHs) exhibited histological features consistent with subacute hemorrhages aged between five and seven days. These included the presence of granulation tissue, erythrocyte degradation, and scattered siderophages. A thin layer of fibroblasts was observed between the dura mater and the clot, which was infiltrated by newly formed capillary blood vessels. Marked edema was evident, characterized by prominent pericellular and perivascular empty spaces.	subdural hemorrhages with midline shift

N/A: not applicable.

## Data Availability

Data sharing is not applicable to this article as no new data were created or analyzed in this study.

## References

[1] Carrigan T.D. (2000). Domestic Violence: The Shaken Adult Syndrome. Emerg. Med. J..

[2] Bauerová A., Nosková P., Bláha J. (2021). Shaken Adult Syndrome or a Neurological Complication of Epidural Anesthesia?. Anest. Intenziv. Med..

[3] Chia J.K.K., Goh K.Y.C., Chan C. (2000). An Unusual Case of Traumatic Intracranial Hemorrhage Caused by Wakeboarding. Pediatr. Neurosurg..

[4] Arneitz C., Schmitz J., Szilagyi I., Kienesberger B., Schalamon G., Senica S.O., Schalamon J. (2024). Abusive Head Trauma and Crying Infant-Public Awareness of Newborn and Infant Trauma. Acta Paediatr..

[5] Feld K., Feld D., Karger B., Helmus J., Schwimmer-Okike N., Pfeiffer H., Banaschak S., Wittschieber D. (2021). Abusive Head Trauma in Court: A Multi-Center Study on Criminal Proceedings in Germany. Int. J. Leg. Med..

[6] Goethals L., Prokofieva Nelson V., Fenouillet F., Chevreul K., Bergerat M., Lebreton C., Refes Y., Blangis F., Chalumeau M., Le Roux E. (2024). Characteristics and Popularity of Videos of Abusive Head Trauma Prevention: Systematic Appraisal. J. Med. Internet Res..

[7] Oates A.J., Sidpra J., Mankad K. (2021). Parenchymal Brain Injuries in Abusive Head Trauma. Pediatr. Radiol..

[8] Drommi M., Barranco R., Ventura F., Molinelli A. (2025). Elder Abuse in Europe’s “Most Elderly” City: An Update of the Phenomenon Based on the Cases Reported to the Penal Court of Genoa from 2020 to 2023 and Literature Review. Aging Clin. Exp. Res..

[9] Jung J.-B., Lee K.M., Park S.H., Park S.-H. (2025). Medicolegal Entomology in an Elder Neglect Investigation: A Case Study from South Korea. Leg. Med..

[10] Santos J.d.S., Almeida F.d.C.A.d., Soares J.d.S., Gomes I.V.d.N.P., Castañeda R.F.G., Souto R.Q. (2025). Forensic interventions carried out by nurses on older people in situations of violence: A comparative study. Rev. Esc. Enferm. USP.

[11] Son Y.-J., Jo N.Y., Lee Y.M., Park S.H. (2025). Development and Psychometric Evaluation of the Forensic Nursing Competency Scale-Short Form for Hospital Nurses. J. Adv. Nurs..

[12] Bugelli V., Campobasso C.P., Feola A., Tarozzi I., Abbruzzese A., Di Paolo M. (2023). Accidental Injury or “Shaken Elderly Syndrome”? Insights from a Case Report. Healthcare.

[13] Pomara C., Bello S., Serinelli S., Fineschi V. (2015). A Rare and Lethal Case of Right Common Carotid Pseudoaneurysm Following Whiplash Trauma. Forensic Sci. Med. Pathol..

[14] Yang L., Liu H., Zhao X., Li H., Zhou D., Wang B., Zhao L., Wang L., Gao Y., Zhu H. (2023). Application of Postmortem MRI for Identification of Medulla Oblongata Contusion as a Cause of Death: A Case Report. Int. J. Leg. Med..

[15] Gill J.R., Goldfeder L.B., Armbrustmacher V., Coleman A., Mena H., Hirsch C.S. (2009). Fatal Head Injury in Children Younger than 2 Years in New York City and an Overview of the Shaken Baby Syndrome. Arch. Pathol. Lab. Med..

[16] Zerbo S., Bilotta C., Perrone G., Malta G., Re G.L., Terranova M.C., Argo A., Salerno S. (2021). Preventable Fatal Injury during Rally Race: A Multidisciplinary Approach. Int. J. Leg. Med..

[17] Sacco M.A., Verrina M.C., Raffaele R., Gualtieri S., Tarallo A.P., Gratteri S., Aquila I. (2025). The Role of Autopsy in the Forensic and Clinical Evaluation of Head Trauma and Traumatic Brain Injury in Road Traffic Accidents: A Review of the Literature. Diagnostics.

[18] Shkrum M.J., Green R.N., Nowak E.S. (1989). Upper Cervical Trauma in Motor Vehicle Collisions. J. Forensic Sci..

[19] Monson K.L., Converse M.I., Manley G.T. (2019). Cerebral Blood Vessel Damage in Traumatic Brain Injury. Clin. Biomech..

[20] Knackstedt H., Kråkenes J., Bansevicius D., Russell M.B. (2012). Magnetic Resonance Imaging of Craniovertebral Structures: Clinical Significance in Cervicogenic Headaches. J. Headache Pain..

[21] Goel A., Blaskovich S., Shah A., Prasad A., Vutha R., Shukla A. (2024). Post-Traumatic Central or Axial Atlantoaxial Dislocation Presenting with “Atypical” Symptoms-Analyzing the Role of Dynamic Imaging on the Basis of Experience with 14 Patients Treated by Atlantoaxial Fixation Surgery. World Neurosurg..

[22] Radanov B.P., Sturzenegger M., De Stefano G., Schnidrig A. (1994). Relationship between Early Somatic, Radiological, Cognitive and Psychosocial Findings and Outcome during a One-Year Follow-up in 117 Patients Suffering from Common Whiplash. Br. J. Rheumatol..

[23] Brum M., Reimão S., Sousa D., de Carvalho R., Ferreira J.J. (2016). Spinal Cord Lesion by Minor Trauma as an Early Sign of Multiple System Atrophy. Front. Neurol..

[24] Silva D.A., de Aguiar G.B., Jory M., Conti M.L.M., Veiga J.C.E. (2020). “Whiplash” Cervical Trauma with Fracture and Migration of Carotid Stent Fragments. Surg. Neurol. Int..

[25] Schoppe C.H., Lantz P.E. (2013). Are Peripapillary Intrascleral Hemorrhages Pathognomonic for Abusive Head Trauma?. J. Forensic Sci..

[26] Ding Y., Lu Q., Wang C.G., Hu Y. (2018). Behavioral Characteristics and Medicolegal Identification of Infanticide. Fa Yi Xue Za Zhi.

[27] Pasquale-Styles M.A., Crowder C.M., Fridie J., Milla S.S. (2014). Bilateral First Rib Anomalous Articulations with Pseudarthroses Mimicking Healing Fractures in an Infant with Abusive Head Injury. J. Forensic Sci..

[28] Sauvageau A., Bourgault A., Racette S. (2008). Cerebral Traumatism with a Playground Rocking Toy Mimicking Shaken Baby Syndrome. J. Forensic Sci..

[29] Porzionato A., Macchi V., Aprile A., De Caro R. (2008). Cervical Soft Tissue Lesions in the Shaken Infant Syndrome: A Case Report. Med. Sci. Law.

[30] Tuchtan L., Lebreton-Chakour C., Tosello B., Oger M., Piercecchi-Marti M.-D., Bartoli C. (2017). Coexistence of Subdural Hematoma and a Rare Cardiopathy in an Infant: Etiological and French Medicolegal Discussion. J. Forensic Sci..

[31] Timonov P., Fasova A., Braynova I., Novakov I., Poryazova E. (2023). Difficulties Encountered by Forensic Pathologists in Proving Abusive Head Trauma in Children: A Case Report. Cureus.

[32] Unuma K., Makino Y., Yamamoto K., Hattori S., Arai N., Sakai K., Kitagawa M., Uemura K., Kanegane H. (2021). Fatal Intracranial Hemorrhage Due to Infantile Acute Lymphoblastic Leukemia Mimicking Abusive Head Trauma. J. Forensic Sci..

[33] Guddat S.S., Ehrlich E., Martin H., Tsokos M. (2011). Fatal Spontaneous Subdural Bleeding Due to Neonatal Giant Cell Hepatitis: A Rare Differential Diagnosis of Shaken Baby Syndrome. Forensic Sci. Med. Pathol..

[34] Deutsch S.A., Teeple E., Dickerman M., Macaulay J., Collins G. (2020). For Victims of Fatal Child Abuse, Who Has the Right to Consent to Organ Donation?. Pediatrics.

[35] Bartschat S., Richter C., Stiller D., Banschak S. (2016). Long-Term Outcome in a Case of Shaken Baby Syndrome. Med. Sci. Law.

[36] Matlung S.E., Bilo R.A.C., Kubat B., van Rijn R.R. (2011). Multicystic Encephalomalacia as an End-Stage Finding in Abusive Head Trauma. Forensic Sci. Med. Pathol..

[37] Di Fazio N., Delogu G., Morena D., Cipolloni L., Scopetti M., Mazzilli S., Frati P., Fineschi V. (2023). New Insights into the Diagnosis and Age Determination of Retinal Hemorrhages from Abusive Head Trauma: A Systematic Review. Diagnostics.

[38] Maiese A., Iannaccone F., Scatena A., Del Fante Z., Oliva A., Frati P., Fineschi V. (2021). Pediatric Abusive Head Trauma: A Systematic Review. Diagnostics.

[39] Gleckman A.M., Kessler S.C., Smith T.W. (2000). Periadventitial Extracranial Vertebral Artery Hemorrhage in a Case of Shaken Baby Syndrome. J. Forensic Sci..

[40] Salvatori M.C., Lantz P.E. (2015). Retinal Haemorrhages Associated with Fatal Paediatric Infections. Med. Sci. Law.

[41] Snow P.C. (2021). Psychosocial Adversity in Early Childhood and Language and Literacy Skills in Adolescence: The Role of Speech-Language Pathology in Prevention, Policy, and Practice. Perspect. ASHA SIGs.

[42] Page M.J., McKenzie J.E., Bossuyt P.M., Boutron I., Hoffmann T.C., Mulrow C.D., Shamseer L., Tetzlaff J.M., Akl E.A., Brennan S.E. (2021). The PRISMA 2020 Statement: An Updated Guideline for Reporting Systematic Reviews. PLoS Med..

[43] Del Duca F., Ghamlouch A., Manetti A.C., Napoletano G., Sonnini E., Treves B., De Matteis A., La Russa R., Sheppard M.N., Fineschi V. (2024). Sudden Cardiac Death, Post-Mortem Investigation: A Proposing Panel of First Line and Second Line Genetic Tests. J. Pers. Med..

[44] Del Duca F., Napoletano G., Volonnino G., Maiese A., La Russa R., Di Paolo M., De Matteis S., Frati P., Bonafè M., Fineschi V. (2024). Blood–Brain Barrier Breakdown, Central Nervous System Cell Damage, and Infiltrated T Cells as Major Adverse Effects in CAR-T-Related Deaths: A Literature Review. Front. Med..

[45] Maiese A., Del Duca F., Ghamlouch A., Treves B., Manetti A.C., Napoletano G., De Matteis A., Dimattia F., Wan H., Pignataro L. (2024). Sudden Death: A Practical Autopsy Approach to Unexplained Mediastinitis Due to Fatal Untreated Neck Infections—A Systematic Review. Diagnostics.

[46] Maiese A., Manetti A.C., Santoro P., Del Duca F., De Matteis A., Turillazzi E., Frati P., Fineschi V. (2023). FOXO3 Depletion as a Marker of Compression-Induced Apoptosis in the Ligature Mark: An Immunohistochemical Study. Int. J. Mol. Sci..

[47] Maiese A., Spina F., Visi G., Del Duca F., De Matteis A., La Russa R., Di Paolo M., Frati P., Fineschi V. (2023). The Expression of FOXO3a as a Forensic Diagnostic Tool in Cases of Traumatic Brain Injury: An Immunohistochemical Study. Int. J. Mol. Sci..

[48] Geddes J.F., Whitwell H.L. (2003). Shaken Adult Syndrome Revisited. Am. J. Forensic Med. Pathol..

[49] Azari A.A., Kanavi M.R., Saipe N.B., Potter H.D., Albert D.M., Stier M.A. (2013). Shaken Adult Syndrome: Report of 2 Cases. JAMA Ophthalmol..

[50] Li Z., Wang J., Zhang J., Jia M., Xu Q., Chen M., Zou D., Ma K., Chen Y. (2022). Cerebral Hemorrhage Caused by Shaking Adult Syndrome? Evidence from Biomechanical Analysis Using 3D Motion Capture and Finite Element Models. Int. J. Leg. Med..

[51] Ninomiya K., Nakaza E., Yamashiro T., Abe T., Ikematsu N., Nagama H., Kakazu K., Fukasawa M. (2023). Shaken Adult Syndrome Due to Ocean Wave: An Autopsy Case. Forensic Sci. Med. Pathol..

[52] Calhoun C.D., Stone K.J., Cobb A.R., Patterson M.W., Danielson C.K., Bendezú J.J. (2022). The Role of Social Support in Coping with Psychological Trauma: An Integrated Biopsychosocial Model for Posttraumatic Stress Recovery. Psychiatr. Q..

[53] Ritchie J., Doherty M. (2025). Medico-Legal Evidence: Survivor Relational Autonomy and Informed Consent in Sexual Assault Examinations. Fem. Leg. Stud..

[54] Peterman A., Devries K., Guedes A., Chandan J.S., Minhas S., Lim R.Q.H., Gennari F., Bhatia A. (2023). Ethical Reporting of Research on Violence against Women and Children: A Review of Current Practice and Recommendations for Future Guidelines. BMJ Glob. Health.

[55] Saternus K.-S., Kernbach-Wighton G., Oehmichen M. (2000). The Shaking Trauma in Infants—Kinetic Chains. Forensic Sci. Int..

[56] Palmieri M., Frati A., Santoro A., Frati P., Fineschi V., Pesce A. (2021). Diffuse Axonal Injury: Clinical Prognostic Factors, Molecular Experimental Models and the Impact of the Trauma Related Oxidative Stress. An Extensive Review Concerning Milestones and Advances. Int. J. Mol. Sci..

[57] Pinchi E., Luigi C., Paola S., Gianpietro V., Raoul T., Mauro A., Paola F. (2020). MicroRNAs: The New Challenge for Traumatic Brain Injury Diagnosis. Curr. Neuropharmacol..

[58] Mills M.D. (1998). Terson Syndrome. Ophthalmology.

[59] Moreno A., Grodin M.A. (2002). Torture and Its Neurological Sequelae. Spinal Cord.

